# Attack of the Clones: Microglia in Health and Disease

**DOI:** 10.3389/fncel.2022.831747

**Published:** 2022-01-31

**Authors:** Amritha Vinayak Manjally, Tuan Leng Tay

**Affiliations:** ^1^Faculty of Biology, University of Freiburg, Freiburg, Germany; ^2^BrainLinks-BrainTools Centre, University of Freiburg, Freiburg, Germany; ^3^Department of Biology, Boston University, Boston, MA, United States; ^4^Freiburg Institute of Advanced Studies, University of Freiburg, Freiburg, Germany; ^5^Department of Anatomy and Neurobiology, Boston University School of Medicine, Boston, MA, United States

**Keywords:** microglia, clonality, development, homeostasis, repopulation, neurodegeneration

## Introduction

Microglia are brain-resident macrophages that carry out immune surveillance, support neurogenesis and neuronal survival, shape the neuronal network, and maintain tissue homeostasis (Nimmerjahn et al., 2005; Hanisch and Kettenmann, 2007; Sierra et al., 2010; Tremblay et al., 2010; Schafer et al., 2012; Ueno et al., 2013; Squarzoni et al., 2014; Schafer and Stevens, 2015; Diaz-Aparicio et al., 2020). The adult resident pool of microglia is primarily derived from yolk sac (YS) erythromyeloid progenitors (EMPs) that have clonally proliferated within the brain parenchyma during development (Alliot et al., 1999; Ginhoux et al., 2010; Hashimoto et al., 2013; Gomez Perdiguero et al., 2015). To maintain their cell density in adulthood, the resident microglial cells undergo local clonal self-renewal (Ajami et al., 2007; Askew et al., 2017; Réu et al., 2017; Tay et al., 2017). A study on parabiotic chimeric mice revealed that the resident microglial population is exclusively replenished by locally derived microglial clones with no evidence of contribution from peripheral myeloid progenitors (Ajami et al., 2007). Clonal expansion of non-ablated residual microglia was also found to be responsible for re-establishing steady state microglial cell densities following a pharmacological ablation (Huang et al., 2018). Homeostatic microglia continuously monitor the brain environment, scavenge dying cells and cellular debris, and rapidly respond to any tissue damage (Kreutzberg, 1996; Streit et al., 1999). In response to acute pathology, resident microglia rapidly accumulate around the lesion via clonal microgliosis (Streit et al., 1999; Ladeby et al., 2005; Ajami et al., 2007; Ransohoff, 2007). Microgliosis at the site of CNS damage has been shown to be governed by signaling molecules like colony-stimulating factor-1 (CSF1), fractalkine receptor (CX3CR1) and purinergic receptor P2Y12 (P2RY12) (Guan et al., 2016; Gu et al., 2016; Peng et al., 2016). To repair damage, microglia elicit proinflammatory cytokines and later transition to anti-inflammatory phenotypes (Colton, 2009; Lloyd et al., 2019). Several studies have suggested that clinical recovery of acute lesions is accompanied by the resolution of proliferated microglia by migration and cell death (Dihné et al., 2001; Wilson et al., 2004; Tay et al., 2017; Lloyd et al., 2019). However, in severe or chronic neurodegenerative pathologies like Alzheimer's disease (AD), Parkinson's disease (PD), multiple sclerosis (MS), and motor neuron diseases, microglial clonal expansion has been persistently observed around lesions and plaques (Glass et al., 2010; Streit et al., 2020). In AD and MS animal models, microglial proliferation around the Aβ plaques and demyelinating neurons was actively promoted by CSF1R and triggering receptor expressed on myeloid cells 2 (TREM2) (Cantoni et al., 2015; Olmos-Alonso et al., 2016; Wang et al., 2016; Jay et al., 2017; Gushchina et al., 2018; Zhao et al., 2018). CSF1R, transforming growth factor beta (TGFβ), and purinergic signaling pathways that influence microglial cell densities are impaired in neurodegenerative diseases (Gómez-Nicola et al., 2013; Von Bernhardi et al., 2015; Olmos-Alonso et al., 2016; Pietrowski et al., 2021). Taken together, the capacity for microglial clonal expansion, proliferation, or renewal, clearly play an important function across CNS development, health, and disease. Although clonal expansion and renewal of microglia appears necessary for physiological brain development, maintenance of CNS health, and response to acute damage, whether microglial clones are beneficial or detrimental in chronic neurodegeneration remains unclear.

**Figure 1:**
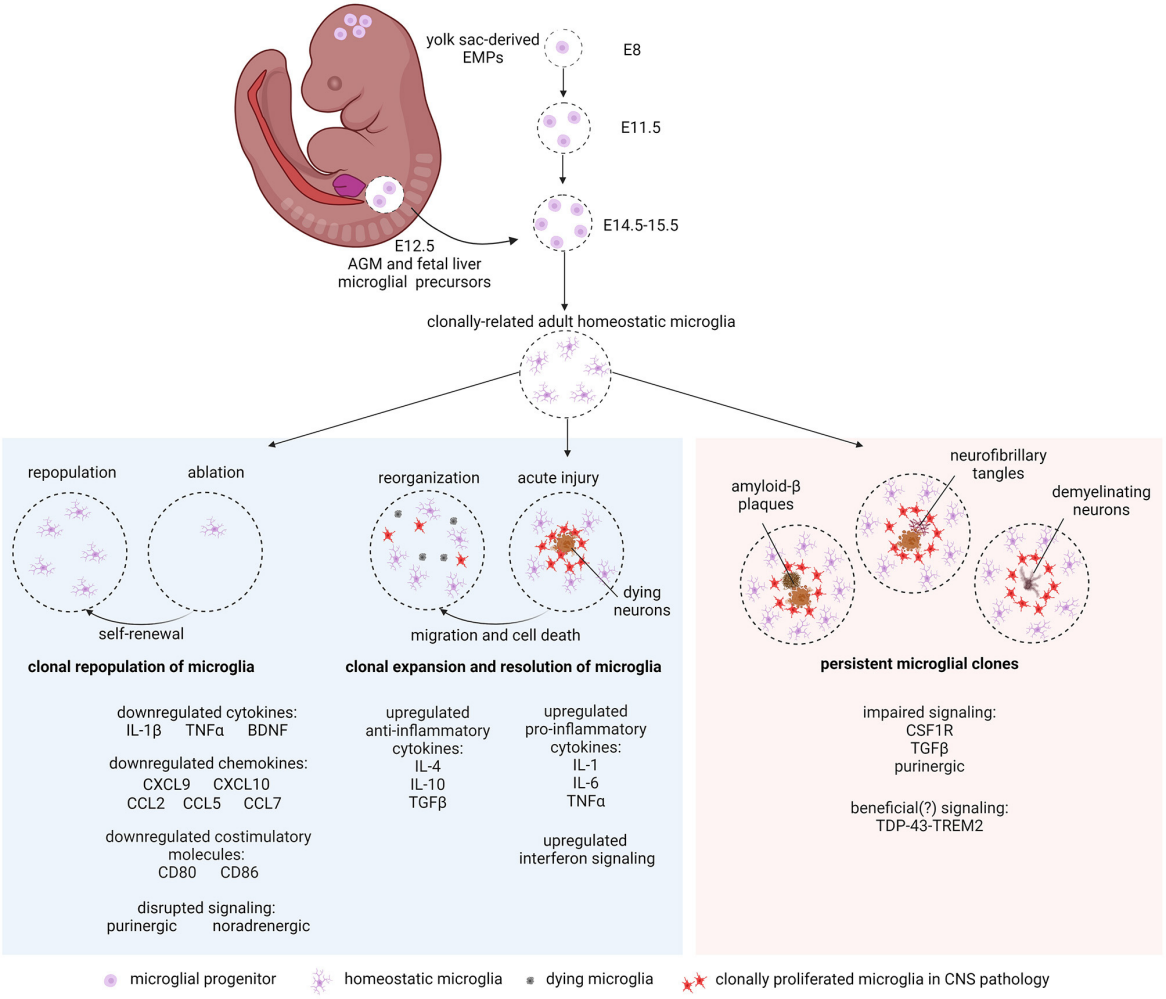
Microglial clonality in development, health, and disease. Yolk sac EMPs enter the mouse neuroepithelium from E8 to E11.5 where they clonally expand and colonize the brain niche. An additional influx of *Hoxb8*^+^ microglial precursors from the AGM and fetal liver at E12.5 invade and populate the brain between E14.5 and E15.5 to make up the rest of the postnatal resident microglial population. Microglia clonally repopulate after ablation via self-renewal (left panel). In acute injury, microglia respond by microgliosis (clonal proliferation) at the site of damage (center panel). With clinical recovery, clones of microglia are reorganized by microglial cell migration and cell death (center panel). In chronic neurodegeneration such as AD, PD and MS, microglial clones persist around Aβ plaques, neurofibrillary tangles (tauopathy), and demyelinating neurons (right panel). Altered gene regulation and signaling pathways mentioned in the text are indicated. Created with BioRender.com.

In this opinion article, we discuss the implications of the formation of microglial clones in health and disease, independent of peripheral myeloid recruitment to the CNS in similar contexts (Figure 1). Several studies have claimed that microglia surrounding plaques and lesions exert detrimental effects and exacerbate disease conditions (Streit et al., 2009; Lassmann et al., 2012; Keren-Shaul et al., 2017; Krasemann et al., 2017; Shahidehpour et al., 2021). Considering that the restoration of tissue homeostasis coincides with the resolution of microglial clones to regain steady state microglial tiling (Dihné et al., 2001; Wilson et al., 2004; Tay et al., 2017; Lloyd et al., 2019), we hypothesize that unresolved microglial clones contribute to the prolongation of neurodegenerative states in chronic neuropathologies. As a hallmark of CNS pathology, microgliosis is likely important to limit tissue damage and infection and for local repair, as is typical in inflammatory responses of tissue-resident macrophages (Jenkins et al., 2011). A timely resolution of excess microglia resulting from clonal expansion is expected to aid or accompany the restoration of homeostasis and clinical recovery. However, sustained presence of reactive microglial clones at high densities around lesions or plaques with no signs of resolution likely lead to neurotoxic outcomes. To explore our hypothesis, we examine various contexts in which microglial clonal expansion has taken place and consider if strategic targeting of microglial clones could ameliorate chronic disease states.

## Microglial Clonality in the Developing and Early Postnatal Brain

Resident microglia of the CNS arise from clones of early EMPs during development. Starting from embryonic day 8 (E8), the first microglial progenitor was observed to migrate from the YS into the neural tube (Alliot et al., 1999). Microglial precursors positive for the F4/80 macrophage marker and proliferating cell nuclear antigen (PCNA) indicated that their active proliferation led to rapid microglial population of the brain parenchyma during embryonic and postnatal stages (Alliot et al., 1999). Fate mapping experiments identified YS EMPs as a common origin for all tissue-resident macrophages from brain microglia to other long-lasting tissue macrophages in the heart, liver, kidney, lungs, and intestines (Ginhoux et al., 2010; Hashimoto et al., 2013; Gomez Perdiguero et al., 2015). Yolk sac-derived microglial progenitors were reported to enter the brain parenchyma in a single wave up to E11.5 and locally proliferate to form the eventual adult resident microglial population (Ginhoux et al., 2010). However, the drastic increase in microglial cell number between E14.5 and E15.5, despite a decline in proliferating microglia, suggested the possibility of a second source of microglial cells that contribute to the resident microglial pool (Swinnen et al., 2013). Notably, genetic targeting of microglial progenitors from the YS have never reached close to 100 % (Ginhoux et al., 2010; Goldmann et al., 2016). *Vav1-Cre*^+^*: dicer*^*fl*/*fl*^ mice were used to study the potential contribution of post-YS definitive hematopoiesis to resident microglia by targeting the microRNA-processing ribonuclease Dicer to deplete any microglia that arise after E11 (Fehrenbach et al., 2018). *Dicer* knock-out in Vav1-expressing hematopoietic cells caused a reduction of ~40% of total IBA1^+^ microglia in comparison to controls at postnatal day (P) 1, suggesting a contribution of post-YS hematopoiesis to the microglial population and/or defective microglial recruitment in the mutants (Fehrenbach et al., 2018). Another interesting finding was the presence of the *Hoxb8*^+^ microglial subpopulation, which makes up ~25% of the total microglial population in the adult cortex (Chen et al., 2010; De et al., 2018). These microglia were found to be of the *Hoxb8* progenitor lineage, which originates in the aorta-gonad-mesonephros (AGM) and fetal liver, where these progenitors selectively proliferate and infiltrate the developing brain at E12.5 (De et al., 2018). Collectively, YS, AGM and fetal liver microglial precursors clonally self-renew to maintain the steady state microglial pool (Figure 1).

Microglia influence early embryonic forebrain development and the wiring of neuronal circuits (Li et al., 2019). At P3, F4/80^+^ microglia appear to play a role in eliminating apoptotic Purkinje cells of the developing cerebellum (Marin-Teva et al., 2004). In the first two postnatal weeks, microglia were described to aid synaptogenesis as well as actively prune synapses and engulf synaptic elements via CR3/C3 and fractalkine signaling (Paolicelli et al., 2011; Schafer et al., 2012; Miyamoto et al., 2016). Defective microglia and axonogenesis in *Cx3cr1*^−/−^, *CR3*^−/−^, and *DAP12*^−/−^ mice suggested that forebrain microglia modulate dopaminergic axonal outgrowth and shape the laminar positioning of neocortical intraneuronal subsets prior to birth (Squarzoni et al., 2014). From P2 to P7, several studies have reported a specific proliferative CD11c^+^ CX3CR1^+^ GPNMB^+^ CLEC7A^+^ microglial subset within the developing white matter that was proposed to support myelination and neurogenesis in an insulin-like growth factor 1-dependent manner (Hagemeyer et al., 2017; Wlodarczyk et al., 2017; Li et al., 2019). The brief appearance of these proliferative white matter-associated microglia temporally aligned with the onset of myelination and the death of ~50% of newly formed oligodendrocytes (Barres et al., 1992; Trapp et al., 1997). This proliferative microglial subset purportedly phagocytoses dead oligodendrocytes to free up extracellular space for developing neurons and regulate homeostasis, suggesting a supportive role of these transient perinatal microglial clones (Li et al., 2019). The over two-fold increase in CD11b^+^ microglia peaked by the second postnatal week, followed by a 50% decline in microglial cell density due to reduced microglial proliferation and increased apoptosis from the third to sixth postnatal week, thereafter achieving steady state numbers (Nikodemova et al., 2015). In summary, microglia are basically clonal derivatives of early macrophage precursors from YS and non-YS hematopoietic sources, resulting in a heterogeneous population of microglia in the brain parenchyma. Furthermore, the various perinatal clonal microglial subsets play a supportive and beneficial role in early neuronal network development and maturation, synapse formation, synaptic pruning, and oligodendrogenesis (Barres et al., 1992; Li et al., 2019; Bennett and Bennett, 2020).

## Adult Microglial Clonal Renewal and Repopulation

The ability of microglia to clonally self-renew in every niche of the brain without recruitment of macrophages from other brain-associated compartments or bone marrow stem cells suggests a certain level of plasticity for microglia to maintain their normal cell population in the CNS (Tay et al., 2017). Adult resident microglia undergo proliferation and apoptosis to maintain steady state cell densities (Hashimoto et al., 2013; Askew et al., 2017). On average, microglia self-renew at a proliferation rate of 0.69% in mice and 2% in humans (Askew et al., 2017). In different brain regions, adult resident microglia were predicted to randomly and completely turnover in every 8, 15, and 41 months in the murine olfactory bulb, hippocampus, and cortex, respectively (Tay et al., 2017). The majority of human cortical microglia have an average lifespan of 4.2 years (Réu et al., 2017). Signaling through the CSF1R and interleukin (IL)-1 receptors were shown to be necessary for the maintenance and self-renewal of the microglial population (Bruttger et al., 2015; Zhan et al., 2019). Furthermore, CX3CR1 signaling appeared to be required for the regulation of homeostatic microglial cell density (Zhan et al., 2014).

Microglial depletion studies by genetic or pharmacological approaches have revealed that residual microglial clones reestablish the complete microglial population by clonal expansion (Figure 1). However, transient removal of microglia from the brain is not without adverse consequences. Genetic ablation of 99% of microglia involved administering diphtheria toxin to mouse lines with the expression of diphtheria toxin receptor regulated by the promoter activity of the *Cx3cr1* gene, later leading to loss of synaptic structural plasticity (Parkhurst et al., 2013). A pharmacological method involving the blockade of CSF1R with an inhibitor also resulted in microglial ablation (Elmore et al., 2014; Spangenberg et al., 2019). Sudden loss of microglia in the adult murine brain creates a dramatic change in the brain environment and a drastic downregulation of certain proinflammatory cytokines (e.g., tumor necrosis factor (TNF)-α and IL-1β), chemokines (CXCL9, CXCL10, CCL2, CCL5, and CCL7), and costimulatory molecules (CD80 and CD86) (Bruttger et al., 2015). Studies have identified glia to be major producers of TNF-α, which is also necessary for maintaining synaptic strength and excitatory synapses (Beattie et al., 2002; Stellwagen and Malenka, 2006). Microglia also produce brain-derived neurotrophic factor (BDNF) that mediates synaptic plasticity and synaptogenesis (Parkhurst et al., 2013; Zhou et al., 2019). Purinergic signaling between microglia and neurons was shown to control neuronal activity and confer neuroprotection (Eyo et al., 2014; Badimon et al., 2020). Cortical two-photon imaging of awake mice revealed that noradrenergic signaling through microglial β2-adrenergic receptors affects microglial process dynamics and surveillance (Liu et al., 2019; Stowell et al., 2019). Therefore, the loss of microglia could disrupt physiological neuronal network functions. Transient microglial ablation using the CSF1R inhibitor PLX5622 showed that the absence of microglia in the adult mouse brain caused odor response deficits in the developing granule cells of the olfactory bulb (Wallace et al., 2020). Post-mortem human homozygous CSF1R mutants revealed that complete absence of microglia was comorbid with underdeveloped corpus callosum and several structural anomalies of the brain (Oosterhof et al., 2019). In contrast to a hereditary lack of microglia, the CNS niche was instantaneously repopulated within a very short span of time after experimental microglial depletion in mouse models, indicating an innate inclination to restore healthy microglial tiling whenever possible (Varvel et al., 2012; Elmore et al., 2014; Han et al., 2017). In the case of partial (80%) microglial depletion, repopulation of microglial cells after ablation was contributed by the remaining internal source of parenchymal microglia (Bruttger et al., 2015; Askew et al., 2017). NF-κB signaling contributes to the restoration of homeostatic microglial cell density achieved through self-renewal, local clonal expansion, and the activation of maturation programs (Zhan et al., 2019). Notably, some microglial ablation studies have shown that peripheral myeloid cells partially repopulate the microglial niche in the absence of CNS irradiation (Varvel et al., 2012; Cronk et al., 2018), potentially also via clonal expansion (Shemer et al., 2018). Altogether, we can infer that in the process of regaining steady state, the CNS does not remain deprived of microglia for long periods in the absence of any interventions to block their survival. This is evident from the instantaneous proliferation and repopulation of these important CNS-resident macrophages by residual microglia. Collectively, clonal renewal and expansion of microglia within the brain parenchyma appear to be necessary and protective against damaging impacts on the CNS, which do not seem to tolerate any prolonged absence of microglia.

## Microglial Clonal Expansion and Resolution In CNS Pathologies

Microgliosis is the active proliferation of microglial cells in response to pathology. This was initially demonstrated in a sciatic nerve injury model, where neuroglia were reported to proliferate in the spinal cord of irradiated rats after sciatic nerve transection (Gilmore, 1975). Following sciatic nerve injury, the increase in anti-CD11b antibody (OX-42)-stained cells in the spinal cord and brain stem areas suggested the proliferation of microglial cells (Eriksson et al., 1993). In other examples, 3 days after peripheral nerve injury, the BrdU-labeled cells that were mostly IBA1^+^ microglia in the injured spinal cord increased by 24 and 11 folds in the dorsal and ventral horn, respectively, in comparison to the intact rat brain (Echeverry et al., 2008). Ten days after intraorbital optic nerve transection, there was a peak in the number of BrdU^+^ F4/80^+^ retinal microglia (Wohl et al., 2010). Yuan and colleagues described a morphologically unique BrdU^+^ IBA1^+^ “rod” microglia that underwent local microgliosis peaking at 7 days following optic nerve transection, without evidence of peripheral monocyte infiltration (Yuan et al., 2015). Clonally expanded Ki67^+^ IBA1^+^ microglia, also indicated by fluorescent Confetti markers, were observed within the injured facial nucleus in the facial nerve axotomy acute neurodegeneration model where the number of microglial cells increased by eight folds (Tay et al., 2017). At 3–5 days post excitotoxic lesions, a high number of IB-4^+^ microglial cells, which were mostly PCNA-positive indicating active proliferation, were found in the ipsilateral substantia nigra pars reticulata (Dihné et al., 2001). Peripheral nerve injury models showed the influence of CSF1R signaling in microgliosis, which is also partially regulated by CX3CR1 and purinergic P2Y12 signaling (Guan et al., 2016; Gu et al., 2016; Peng et al., 2016). Following acute demyelination in the mouse spinal cord, single-cell RNA-sequencing (scRNAseq) revealed a subpopulation of microglia with an activated profile characterized by an increase in interferon signaling (Plemel et al., 2020).

In acute CNS pathologies, clonally expanded microglial populations were observed to resolve (Figure 1). Clinical recovery is accompanied by the reorganization of accumulated microglial clones in the area of injury or damage. In demyelination mouse models based on parabiosis and white matter demyelinating lesions, a transition from proinflammatory to an increasingly anti-inflammatory microglial cell population occurs at the initiation of remyelination in the white matter regions (Miron et al., 2013). Microglial subsets near the lesion sites displayed proinflammatory phenotypes such as high levels of expression of cytokines, including IL1 (specifically IL1β), IL-6, and TNFα (Basu et al., 2002; Li et al., 2003; Yang et al., 2004; Sato et al., 2012). In the lysophosphatidylcholine-induced demyelination mouse model, the transition from a proinflammatory to a pro-regenerative microglial phenotype is accompanied by the necroptosis of proinflammatory microglia before the onset of remyelination (Lloyd et al., 2019). By 10 days post-lesion, pro-regenerative microglia upregulate genes associated with remyelination (e.g., *Matn2, Osm, Fgf1*, and *Cd300lf* ) and myelination (e.g., *Bmp1, Cd69*, and *Fabp5*) (Lloyd et al., 2019). In contrast to proinflammatory phenotypes, microglia transition to express anti-inflammatory cytokines such as IL-4, IL-10, and TGFβ, to suppress inflammation and elicit neuroprotective effects (Park et al., 2007; Colton, 2009; Fenn et al., 2014; Lively et al., 2016; Zöller et al., 2018). Following 10 days of excitotoxic lesion, a high number of previously IB-4^+^ PCNA^+^ microglial cells in the ipsilateral substantia nigra pars reticulata appeared to be apoptotic as indicated by TUNEL staining (Dihné et al., 2001). Apoptotic microglia were also observed around 12 months post-trauma in human head injury patients (Wilson et al., 2004). Corresponding to clinical recovery after facial nerve axotomy, some microglia near the lesion in the facial nucleus underwent apoptosis, or were removed by cell migration during the re-establishment of steady state microglial tiling and morphology (Tay et al., 2017). Concomitant with the onset of recovery, bulk RNAseq and scRNAseq analyses revealed the differential regulation of genes implicated in lipid mediation and cell migration, whereas genes belonging to the homeostatic microglial signature such as *Cst3* and *Sparc* were downregulated (Tay et al., 2017, 2018). Taken together, we can infer the following: microglial clones resulting from local microgliosis at an acute lesion site respond to changes in the brain milieu by eliciting pro- and anti-inflammatory molecules required for tissue repair. While the specific mechanisms driving the migration and cell death of excess microglia resulting from clonal expansion are unclear, the phenomena suggest the tendency for microglia to reorganize and restore their homeostasis alongside clinical recovery.

## Microglial Clones Persist in CNS Pathologies

In chronic neurodegeneration, persistent microglial clones can be seen accumulating at lesions and plaques (Figure 1), unlike CNS pathologies discussed above where clinical recovery accompanies the resolution of excess microglial cells. Microgliosis is consistently observed in chronic CNS pathologies. This could also be characterized by local microglial recruitment to the lesion followed by clonal proliferation in disease affected brain areas (Davalos et al., 2005; Füger et al., 2017). In both murine experimental autoimmune encephalomyelitis (EAE) mouse model and human MS pathology, the increase in microglial cell numbers was a result of local microgliosis around demyelinating lesions and dying neurons (Ajami et al., 2011; Zrzavy et al., 2017; Jordão et al., 2019). There is evidence for Ki67^+^ IBA1^+^ microglial clonal expansion in the temporal cortex of human AD brains (Gómez-Nicola et al., 2013; Olmos-Alonso et al., 2016). A progressive increase in IBA1^+^ BrdU^+^ microglia was found near amyloid-β (Aβ) plaques, suggesting microglial clonal expansion in APP/PS1 mice (Olmos-Alonso et al., 2016).

Further studies have demonstrated that microglia located near lesions exhibit a dysfunctional phenotype in chronic pathologies. K-means clustering of the transcriptomic data obtained from FCRLS^+^ microglia in wildtype aging and disease mouse models including SOD1G93A, APP-PS1, and EAE, showed two major gene clusters (Krasemann et al., 2017). One cluster displayed the loss of 68 homeostatic microglial genes that included *Csf1r, Cx3Cr1, Gpr34, Hexb, Mertk, Olfml3, P2ry12, Tgfbr1, Tgfb1*, and *Tmem119*, together with a loss of transcription factors such as *Mef2a, Mafb, Jun, Sall1*, and *Egr1*. The other cluster showed the upregulation of 28 inflammatory molecules, including *Spp1, Itgax, Axl, Lilrb4, Clec7a, Ccl2, Csf1*, and *Apoe*, among which *Apoe* was most upregulated (Krasemann et al., 2017). CLEC7A^+^ P2RY12^−^ microglia that specifically associated with neuritic Aβ plaques were named microglial neurodegenerative phenotype (“MGnD”) by the authors, in contrast to “M0-homeostatic” adult microglia. The authors proposed that the transition from “M0-homeostatic” to “MGnD” was closely linked to neuritic dystrophy, and that the switch was regulated by TREM2-APOE signaling (Krasemann et al., 2017). Single-cell RNAseq studies showed a disease-associated microglial (“DAM”) subset localized near AD plaques that downregulated P2RY12/P2RY13, CX3CR1, and TMEM119 (Keren-Shaul et al., 2017). In cases of AD, dementia with Lewy bodies, as well as limbic-predominant age-related TDP-43 encephalopathy, neuropathological change associated strongly with dystrophic microglia with altered iron homeostasis, rather than with hypertrophic microglia (Shahidehpour et al., 2021). Chronic microglial activation caused oxidative bursts that led to the progression of MS (Lassmann et al., 2012). Reactive microglial populations that upregulate Il-1α, TNF or C1q were shown to promote the neurotoxic phenotype of astrocytes *in vitro* (Liddelow et al., 2017). This observation was recapitulated in disease affected areas of postmortem AD, PD, MS, and amyotrophic lateral sclerosis (ALS) human tissues, in which C3^+^ GFAP^+^ S100β^+^ reactive astrocytes were abundant (Liddelow et al., 2017). Furthermore, scRNAseq revealed the upregulation of TNF in late-response microglial populations found in the hippocampus of *CK-p25* mice in late stages of neurodegeneration (Mathys et al., 2017). The authors proposed that these microglia could be programmed to transition into a neurotoxic population during later stages of pathology, as they were found to upregulate this cytokine already at an early stage of neurodegeneration (Mathys et al., 2017). Another deleterious impact of microglial populations in AD was demonstrated in a mouse model, where Aβ-carrying microglial cells were claimed to induce Aβ plaques in healthy tissue (d'Errico et al., 2021). *In vivo* imaging of *Irf8*^+/+^ *Cx3cr1*^+/−^ *5xFAD* mice showed that motile microglia transported Aβ in response to laser-induced focal tissue injury, leading to the build-up of Aβ plaques at lesion sites. Migration of microglia containing Aβ from old *Cx3cr1*^+/−^ *5xFAD* brain slice to healthy young wildtype brain tissue further argues for the active involvement of microglia in disease propagation (d'Errico et al., 2021). Altogether, these studies point toward detrimental effects of microglial subpopulations in promoting and propagating chronic neurodegenerative diseases. On the contrary, several scRNAseq studies revealed that among lesion-associated microglia, a subset of microglial cells expressed genes that are associated with the homeostatic phenotype (Keren-Shaul et al., 2017; Mathys et al., 2017; Tay et al., 2018). Interestingly, not all microglia that surround lesions contribute to the aggravation of pathology. It is currently unclear if microglia expressing “MGnD”, “DAM”, and similar non-homeostatic microglial phenotypes could be clonally related, or if clonally expanded microglia display transcriptomic heterogeneity in response to their microenvironment. Instead of naming microglia as the major contributors to chronic neuroinflammation, several studies have shown that microglia surrounding the Aβ plaques form a compact physical barrier around them to limit further local neuronal damage (Condello et al., 2015; Yuan et al., 2016; Spangenberg et al., 2019). Moreover, microglial TREM2-mediated clearance of protein aggregates of human TAR-DNA binding protein 43 kDa (TDP-43) was neuroprotective in mouse models for the motor neuron degenerative disease ALS (Spiller et al., 2018; Xie et al., 2021). Taken together, microglial subpopulations near lesions or plaques can potentially have both beneficial and detrimental effects in chronic neurodegeneration. We speculate that it is the persistence of unresolved reactive and proinflammatory microglial clones that aggravates disease and prevents recovery.

## Discussion

Considering microglial clonality in CNS development and homeostasis, we can conclude that the presence of adult microglia is the result of clonally expanding microglial progenitors that colonize the brain parenchyma during development. Based on the rapid repopulation and re-establishment of homeostatic microglia by clonal expansion of residual cells after ablation, it appears that local sources of CSF1R signaling are sufficient to drive the restoration of homeostatic microglial tiling. In acute CNS diseases, clinical recovery coincides with the resolution of microglial clones that had resulted from microgliosis near the sites of damage. On the other hand, in severe chronic neurodegenerative diseases, prevalent microgliosis with no signs of resolution of excess proinflammatory microglia are observed alongside altered CSF1R, TGFβ, and purinergic signaling that influence microglial cell densities and their homeostatic gene signature (Gómez-Nicola et al., 2013; Von Bernhardi et al., 2015; Olmos-Alonso et al., 2016; Zöller et al., 2018; Pietrowski et al., 2021). While current literature does not describe any ongoing resolution of microglial clones near chronic lesions in diseased brains, this does not exclude the low-level existence of resolving microglial clones around sites of neurodegeneration. To reveal such resolution events, we propose approaches similar to partial targeting of microglial cells by Confetti labeling to spatially and temporally address the clonality and resolution of microglia in diseases (Tay et al., 2017). We hypothesize that unresolved microglial subpopulations, in particular those carrying a proinflammatory phenotype and surrounding the lesions over prolonged periods promote neuroinflammation, exacerbate disease conditions, and impede recovery. An alternative explanation is that these microglial subpopulations become dysfunctional, fail to overcome the complex microenvironment of the disease, and do not transition from an adverse proinflammatory state to a pro-regenerative phenotype. To investigate if partial amelioration of disease could be achieved by disrupting microglial clonal expansion in different disease contexts, streamlined strategies to selectively or transiently target, for example, CSF1R, TGFβ or purinergic signaling could be tested (Gómez-Nicola et al., 2013; Olmos-Alonso et al., 2016; Easley-Neal et al., 2019; Obst et al., 2020; Pons et al., 2020; Pietrowski et al., 2021). Finally, while microglia are clonally-related due to their YS, AGM, and fetal liver hematopoietic origins, we acknowledge their fascinating heterogeneity in CNS development, aging and diseases that would make for another interesting review (Grabert et al., 2016; Matcovitch-Natan et al., 2016; Keren-Shaul et al., 2017; Mathys et al., 2017; Tay et al., 2018; Hammond et al., 2019).

## Author Contributions

AVM wrote the manuscript and designed the figure. TLT supervised the project and extensively revised the manuscript. All authors contributed to the article and approved the submitted version.

## Funding

TLT was supported by the Klaus Tschira Boost Fund (KT-10), FRIAS Junior Fellowship, University of Freiburg Research Innovation Fund, Wissenschaftliche Gesellschaft Freiburg (Helmut Holzer Prize), German Research Foundation (EXC 1086), and Ministry of Economics, Science and Arts of Baden-Württemberg (Sustainability Program for Projects of the Excellence Initiative II).

## Conflict of Interest

The authors declare that the research was conducted in the absence of any commercial or financial relationships that could be construed as a potential conflict of interest.

## Publisher's Note

All claims expressed in this article are solely those of the authors and do not necessarily represent those of their affiliated organizations, or those of the publisher, the editors and the reviewers. Any product that may be evaluated in this article, or claim that may be made by its manufacturer, is not guaranteed or endorsed by the publisher.
